# Continuous stimulation of dual-function peptide PGLP-1-VP inhibits the morbidity and mortality of NOD mice through anti-inflammation and immunoregulation

**DOI:** 10.1038/s41598-021-83201-4

**Published:** 2021-02-11

**Authors:** Huashan Gao, Qian Zhao, Shanshan Tang, Kaiying Li, Fujian Qin, Ziwei Song, Yi Pan, Liang Jin, Yanfeng Zhang

**Affiliations:** 1 State Key Laboratory of Natural Medicines, Jiangsu Key Laboratory of Drug Screening, School of Life Science and Technology, China Pharmaceutical University, Nanjing, China; 2 College of Medicine, Pingdingshan University, Pingdingshan, China; 3 Sanquan College of Xinxiang Medical University, Xinxiang, Henan China

**Keywords:** Immunology, Diseases, Endocrine system and metabolic diseases, Immunological disorders, Metabolic disorders

## Abstract

Multiple animal and human studies have shown that administration of GLP-1RA can enhance β-cell recovery, reduce insulin dosage, reduce HbA1c content in the blood, reduce the risk of hypoglycemia and reduce inflammation. In the NOD mouse model, peptide VP treatment can prevent and treat type 1 diabetes through immunomodulation. Therefore, we designed a new dual-functional PGLP-1-VP, which is expected to combine the anti-inflammatory effect of PGLP-1 and the immunomodulatory effect of VP peptide. In streptozotocin-induced hyperglycemic mice model, we demonstrated that PGLP-1-VP can act as a GLP-1R agonist to improve hyperglycemia and increase insulin sensitivity. In the NOD mouse model, PGLP-1-VP treatment reduced morbidity, mortality, and pancreatic inflammation, and showed superior effect to PGLP-1 or VP treatment alone, confirming that PGLP-1-VP may act as a dual-function peptide. PGLP-1-VP provided immunomodulatory effect through increasing Th2 cell percentage and balancing the ratio of Th2/Th1 in spleen and PLN, similar to P277 and VP. Additionally, PGLP-1-VP and PGLP-1 act the anti-inflammation by increasing Treg cells and TGF-β1 content like DPP-IV inhibitor. Taken together, our data shows that the dual-functional PGLP-1-VP reduces morbidity and mortality in the NOD model, suggesting a potential role in preventing and treating type 1 diabetes.

## Introduction

Type 1 diabetes (T1DM) is an autoimmune disease caused by immune-mediated destruction of insulin-producing β cells in the pancreas. Certain subclass of T lymphocytes are involved in executing autoimmune attacks^1–3^, such as Th1 and Th17 CD4+ T cells which play a key role in eliciting an immune response against pancreatic β cells^4–6^. The main treatment of T1DM is daily injection of recombinant insulin. However, exogenous delivery of insulin does not include C-peptide, a natural byproduct formed when proinsulin is cleaved to release insulin. C-peptide is known to provide protective benefits to neurons, microvasculature, and kidney^7–9^. Usually, at the time of diagnosis, the majority of patients with T1DM have 20–30% of preserved β-cell mass^10, 11^, and about 5% of beta cells are still alive 5 years after diagnosis of T1DM^12^.

Glucagon like peptide-1 receptor agonists (GLP-1 RAs) are commonly used to manage type 2 diabetes mellitus (T2DM) due to their ability to enhance glucose-dependent insulin secretion, reduce glucagon secretion, increase β-cell mass, delay gastric emptying and increase satiety^13–16^. Although GLP-1 RAs is not currently used clinically to treat type 1 diabetes, multiple animal and human studies have suggested their potential benefits^17, 18^, such as enhancing recovery of β cells^19^, reducing insulin dose^20^, decreasing HbA1c, lowering risk of hypoglycemia^21^, and resisting inflammation^22^. However, the reported anti-inflammatory effect is due to the DPP-4 inhibitor treatment^23, 24^, at present it is not reported whether GLP-1RAs has anti-inflammatory effect in the aspect of diabetes in vivo except a report in vitro^25^.

P277, a peptide of Hsp60 protein, was reported to induce Th2 response and promotes the production of anti-inflammatory cytokines^26^. However, other reports also reported potential side effects, including that the administration of Hsp60 induced vascular endothelial cell damage^27^, and immunization with P277 also induced atherosclerosis in high cholesterol diet-fed rabbits^28^ and vascular leak syndrome in C57BL/6 mice^29^. We therefore constructed a novel peptide VP based on P277 that did not have these atherogenic side-effects^30^.

Our lab discovered that combining P277 with other proteins or peptides treatment greatly enhanced the therapeutic benefits in NOD mice^31–34^. In our previous study, we designed a GLP-1 analog PGLP-1 which reduced blood glucose, increased β cells mass, and inhibited weight loss in STZ-induced hyperglycemia mice^35^. In the current study, we combined PGLP-1 and VP to design a new fusion peptide PGLP-1-VP, and with the hypothesis that PGLP-1-VP had better therapeutic effect than treatment with PGLP-1 or VP alone.

## Results

### Bioactivity and stability analysis of PGLP-1-VP in vitro

**Figure 1:**
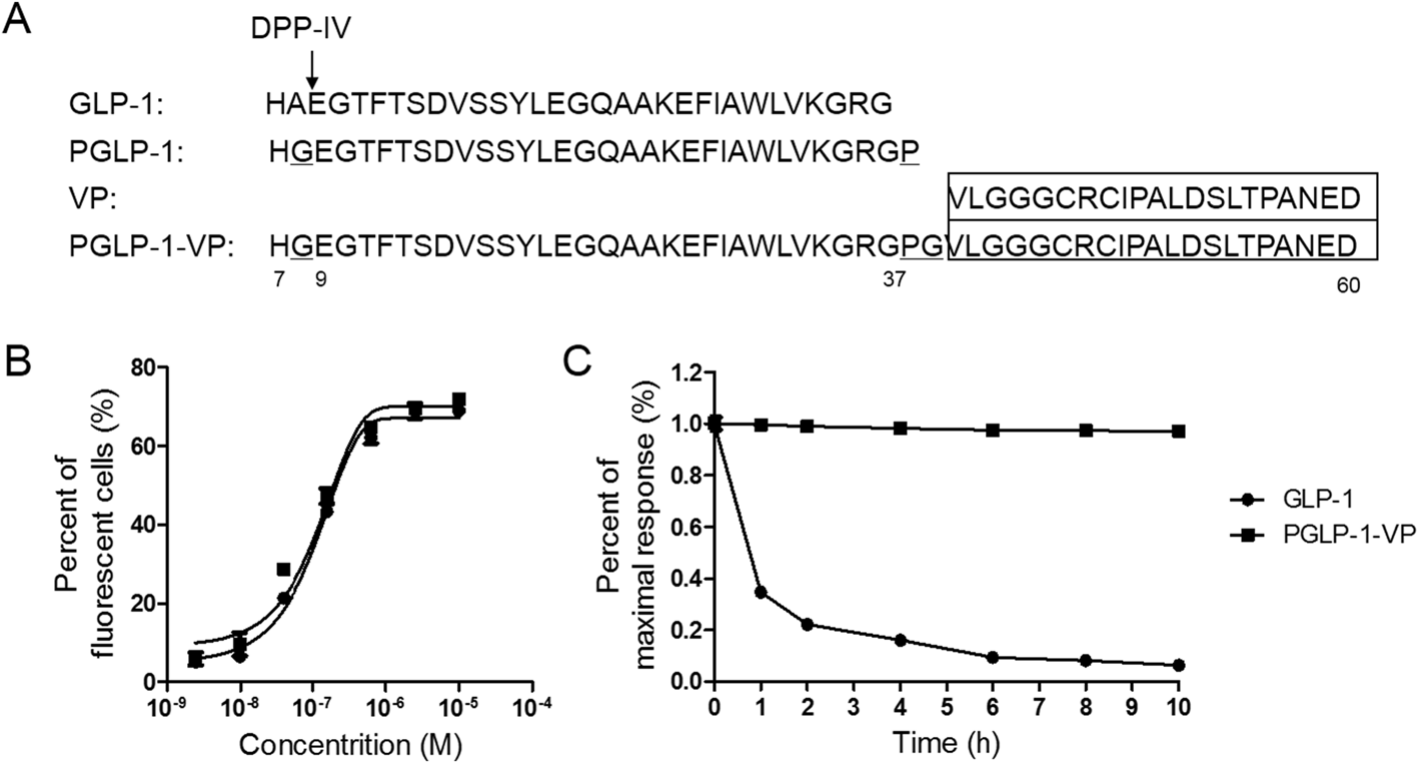
Biological activity of PGLP-1-VP in vitro. (**A**) Sequences of GLP-1, VP, PGLP-1, PGLP-1-VP. (**B**) Dose–response curves for activation of human GLP-1R by GLP-1 or PGLP-1-VP. (**C**) PGLP-1-VP and native GLP-1 against DPP-IV degradation.

PGLP-1-VP is designed as a dual-functional peptide as shown in Fig. 1A. Since the peptide VP was added at the end of the PGLP-1, the ability of the PGLP-VP to activate the GLP1-1R was tested in vitro. PGLP-1-VP was able to activate GLP-1R in a dose-dependent manner (Fig. 1B) and had an equal EC50 compared to GLP-1 (139 ± 37 nmol/L vs 142 ± 32 nmol/L, no significant difference). HPLC was used to evaluate the stability of PGLP-1-VP against DPP-IV degradation in vitro^36^. PGLP-1-VP and GLP-1 were incubated with DPP-IV enzyme at 37 ℃ for 1–10 h and the ratio of remaining intact peptides was examined. After incubating for 10 h, only 6.3% of GLP-1 remained intact. PGLP-1-VP, in contrast, was highly resistant to DPP-IV degradation, with almost 100% of PGLP-1 intact after 10 h of incubation (Fig. 1C).

### Glucose-lowering effect of PGLP-1-VP in normal C57BL/6J mice

**Figure 2:**
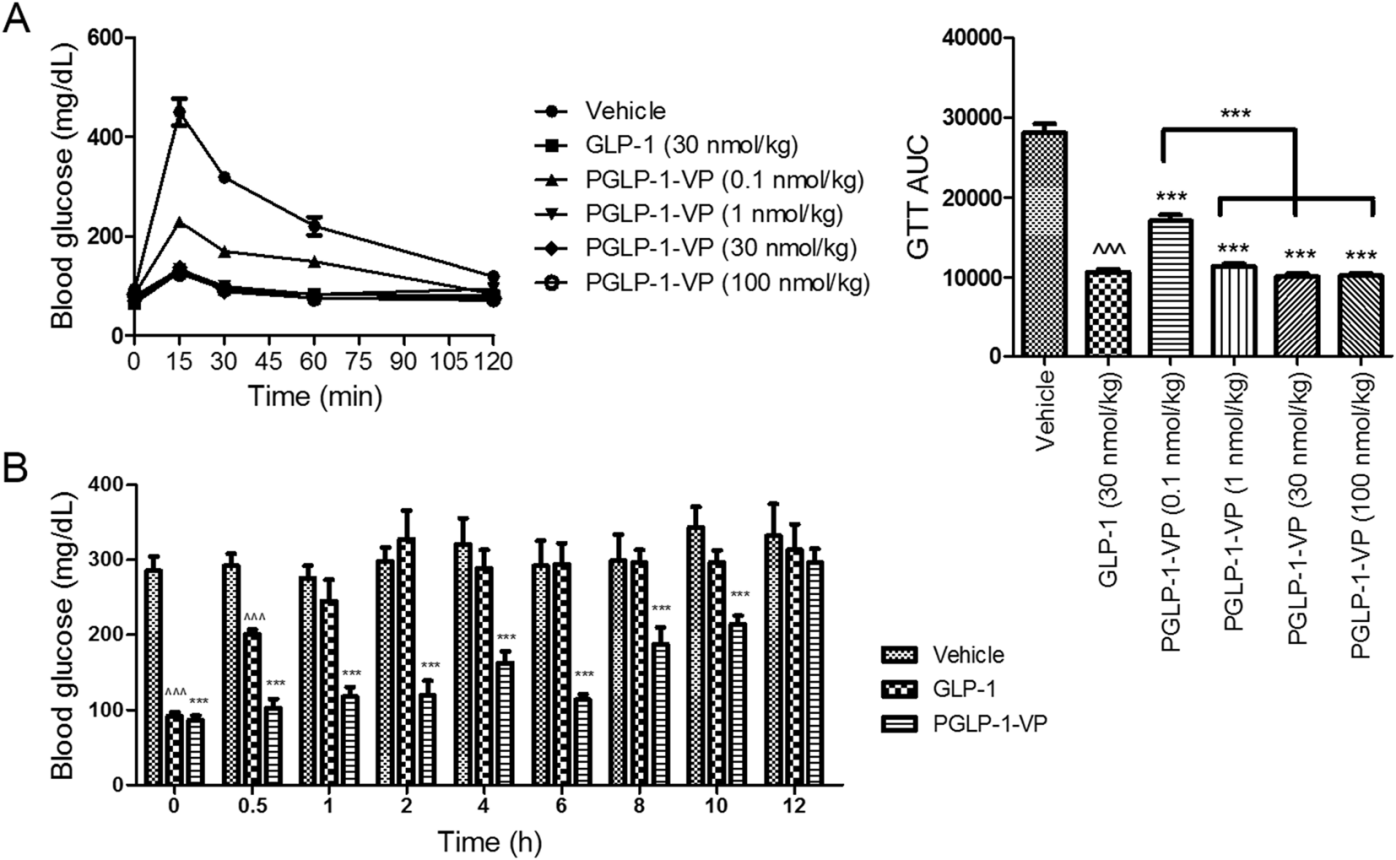
Acute and sustained glucose-lowering effect of GLP-1 or PGLP-1-VP in male C57BL/6J mice (age 8 weeks; n = 6 mice per group). (**A**) Intraperitoneal glucose tolerance test (IPGTT) following a single injection of placebo (Vehicle, PBS), GLP-1 (30 nmol/kg) or PGLP-1-VP (O.1, 1, 30, 100 nmol/kg). (**B**) Blood glucose levels (during an IPGTT, time = 15 min) at different times after subcutaneous administration of placebo (Vehicle, PBS), GLP-1 (30 nmol/kg) or PGLP-1-VP (30 nmol/kg). ***p < 0.001, PGLP-1-VP compared with vehicle. ^^^p < 0.001, GLP-1 compared with vehicle. *GTT* glucose tolerance test, *AUC* area under the concentration time curve.

PGLP-1-VP was able to activate GLP-1R and resist DPP-IV degradation in vitro. We next investigated whether PGLP-1-VP activated GLP-1R in vivo through acute glucose-lowering effect in normal C57BL/6J mice. Subcutaneous injection of 30 nmol/kg of either PGLP-1-VP or GLP-1 both decreased blood glucose and had similar glucose tolerance test area under the curve (GTT AUC) (Fig. 2A). Subcutaneous injection of PGLP-1-VP at 0.1, 1, 30 or 100 nmol/kg also demonstrated dose-dependent glucose-lowering effect in vivo and the minimum effective concentration was 0.1 nmol/kg (Fig. 2A). These results showed that PGLP-1-VP was able to activate GLP-1R and decrease blood glucose in vivo.

To evaluate whether PGLP-1-VP had persistant glucose-lowering effect in vivo, a single dose of GLP-1 (30 nmol/kg), PGLP-1-VP (30 nmol/kg) or placebo was subcutaneously administered, and the blood glucose was measured at pre-determined time intervals. The glucose-lowering effect of PGLP-1-VP was still evident 10 h after administration (Fig. 2B). In contrast, the glucose-lowering effect of native GLP-1 lasted less than 1 h, confirming that PGLP-1-VP was able to work as a long-acting GLP-1R agonist to reduce blood glucose.

### PGLP-1-VP ameliorates STZ-induced hyperglycemia and improves insulin sensitivity

**Figure 3:**
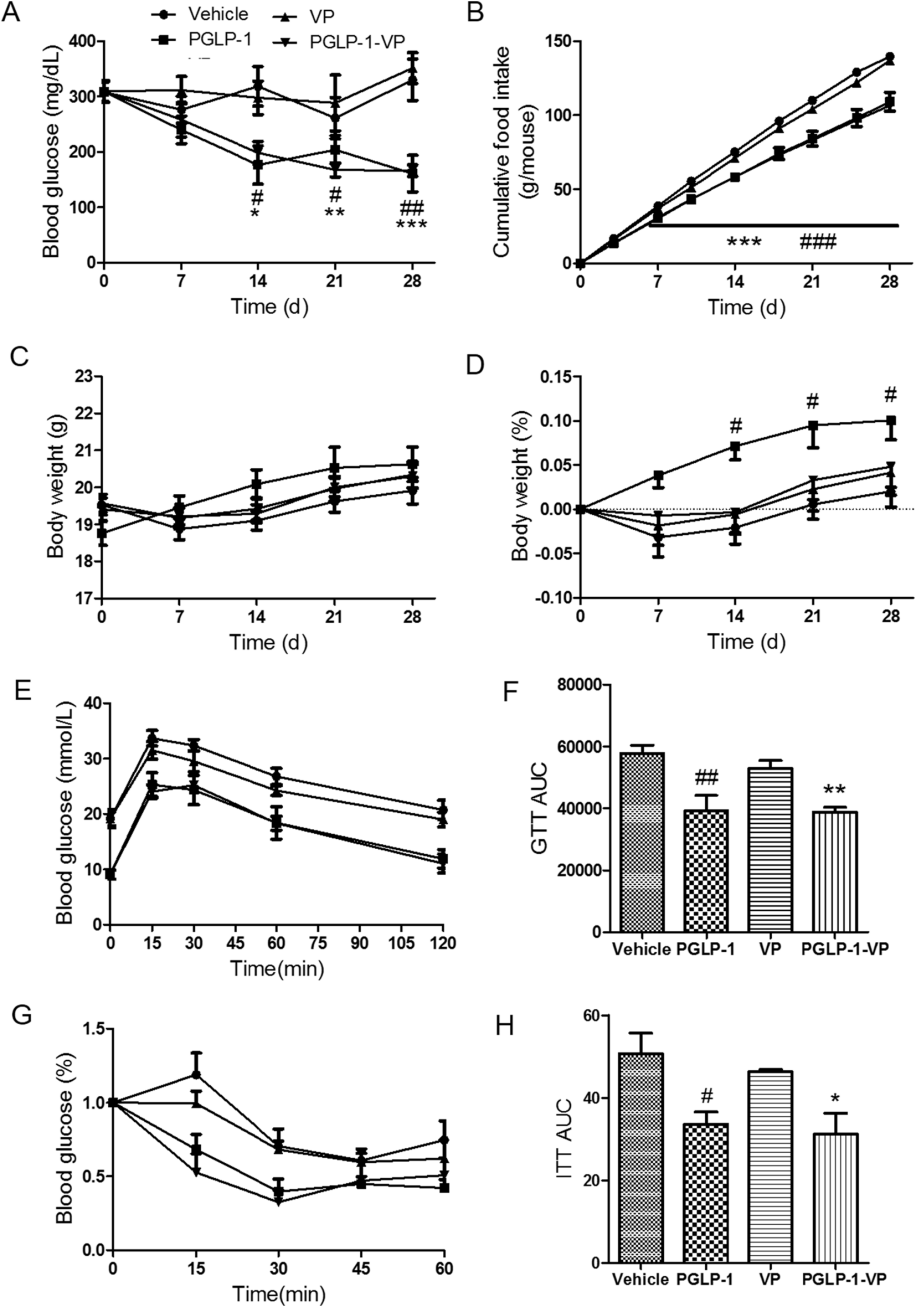
Chronic effect of PGLP-1, VP and PGLP-1-VP on blood glucose, food intake and body weight after 4 weeks treatment. Subcutaneous treatment with PGLP-1, VP, PGLP-1-VP or placebo (2 × 30 nmol/kg/day) in STZ-induced hyperglycemic mice (n = 10 mice per group). (**A**) Weekly measurements of fasted blood glucose. (**B**) Cumulative food intake. Weekly fasted body weight (**C**), Δbody weight (**D**). (**E**,**F**) Week 4, Glucose tolerance tests (GTT) and area under the curve (AUC) evaluation. (**G**,**H**) Insulin tolerance tests (ITT) normalized for basal BG and area under the curve (AUC) evaluation. *p < 0.05, **p < 0.01 and ***p < 0.001, PGLP-1-VP compared with vehicle. ^#^p < 0.05, ^##^p < 0.01, PGLP-1 compared with vehicle.

STZ-induced hyperglycemic mice were treated with placebo, PGLP-1, VP or PGLP-1-VP for 4 weeks. After 2 weeks of treatment, both PGLP-1-VP and PGLP-1 significantly decreased fasting blood glucose compared with vehicle group (p < 0.05). After 4 weeks, fasting blood glucose of PGLP-1-VP and PGLP-1 were less than 200 mg/dL, respectively, 166 mg/dL and 160 mg/dL (PGLP-1-VP: p < 0.001; PGLP-1: p < 0.01, compared with vehicle group) (Fig. 3A). Both PGLP-1-VP and PGLP-1 administration also significantly reduced food intake starting from the second week (PGLP-1-VP or PGLP-1: p < 0.001, compared with vehicle group) (Fig. 3B). However, PGLP-1-VP treatment did not prevent body weight loss, which was significantly inhibited by PGLP-1 treatment (Fig. 3C,D). VP treatment did not decrease fasting blood glucose, inhibit food intake, or inhibit body weight loss compared with vehicle group.

Because PGLP-1 treatment can also significantly improve insulin sensitivity^35^, we next examined whether PGLP-1-VP treatment had a similar function. PGLP-1-VP treatment significantly improve glucose and insulin tolerance in STZ-induced hyperglycemic mice (Fig. 3E–H). GTT AUC showed that both PGLP-1 and PGLP-1-VP had smaller AUC compared with vehicle group (PGLP-1 or PGLP-1-VP: p < 0.01, compared with vehicle group) (Fig. 3F). PGLP-1 and PGLP-1-VP also had a smaller insulin tolerance test area under the curve (ITT AUC) compared with vehicle group (PGLP-1 or PGLP-1-VP: p < 0.05, compared with vehicle group) (Fig. 3H). There was no significant difference between PGLP-1 and PGLP-1-VP. VP treatment did not improve insulin sensitivity. All these data indicated that PGLP-1-VP, as a long GLP-1R agonist, was able to decrease fasting blood glucose, inhibit food intake and improve insulin sensitivity in STZ-induced hyperglycemic mice.

### PGLP-1-VP treatment increases β-cell area

**Figure 4:**
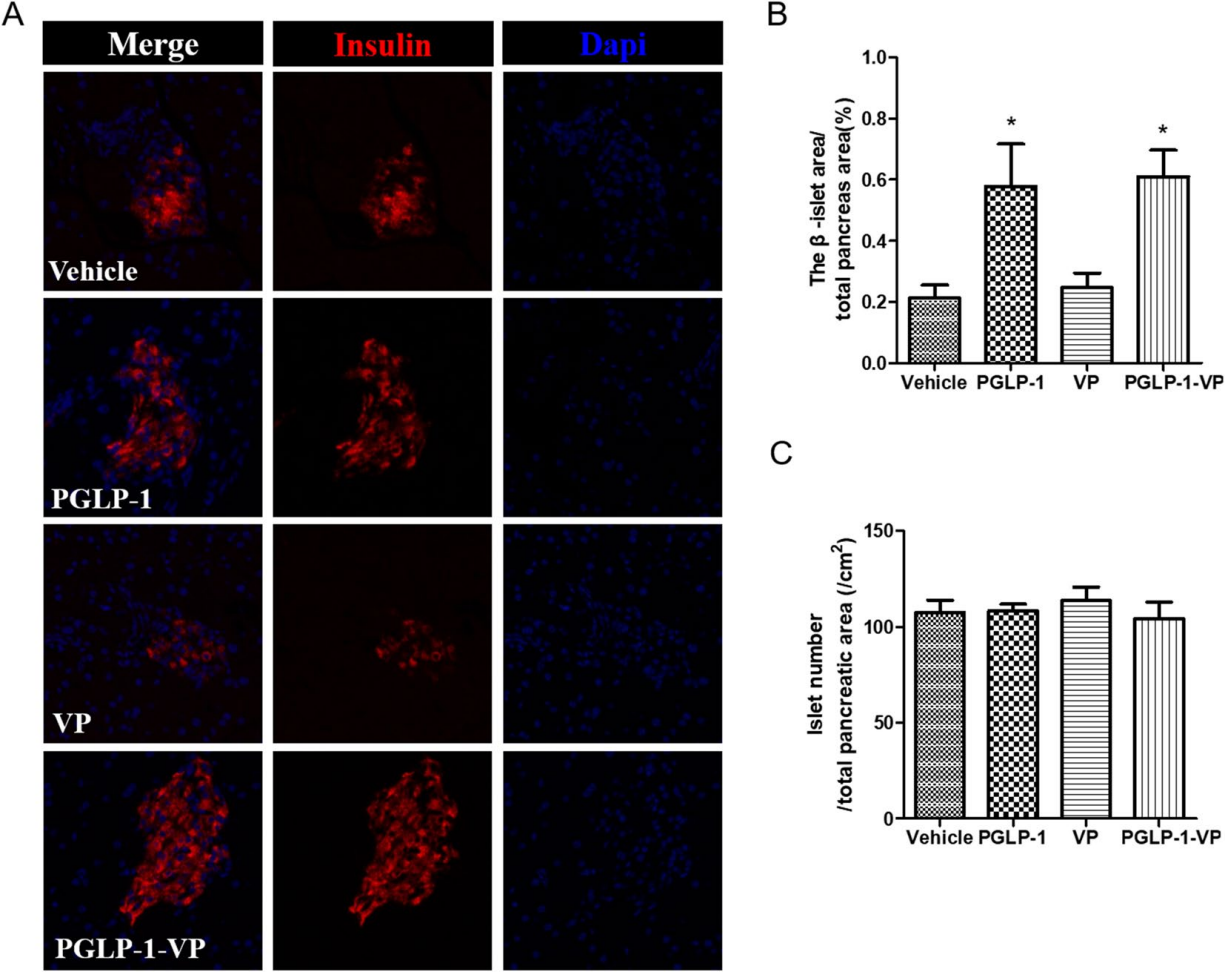
Pancreatic immunoassay in hyperglycemic C57BL/6J mice after 5-week treatment. (**A**) Pancreatic β-cell immunostaining; (**B**) analysis of β-cell area in pancreas (10/10); and (**C**) analysis of islet number in the pancreas.

GLP-1 can increase β-cell mass by stimulating β-cell proliferation and inhibiting apoptosis^14, 15^. In order to assess whether β-cell mass increased, insulin immunofluorescence staining was performed. The results revealed that pancreatic islets β-cells of the Vehicle and VP group was significantly decreased compared to the PGLP-1 and PGLP-1-VP groups (Fig. 4a). Islet β-cells immunofluorescence analysis showed that β-cells area was increased by twofold in the PGLP-1 and PGLP-1-VP groups compared with the Vehicle and VP group (Fig. 4b). There was no significant difference in the total number of islets between the groups (Fig. 4c).

### PGLP-1-VP treatment reduces morbidity and mortality in NOD Mice

**Figure 5:**
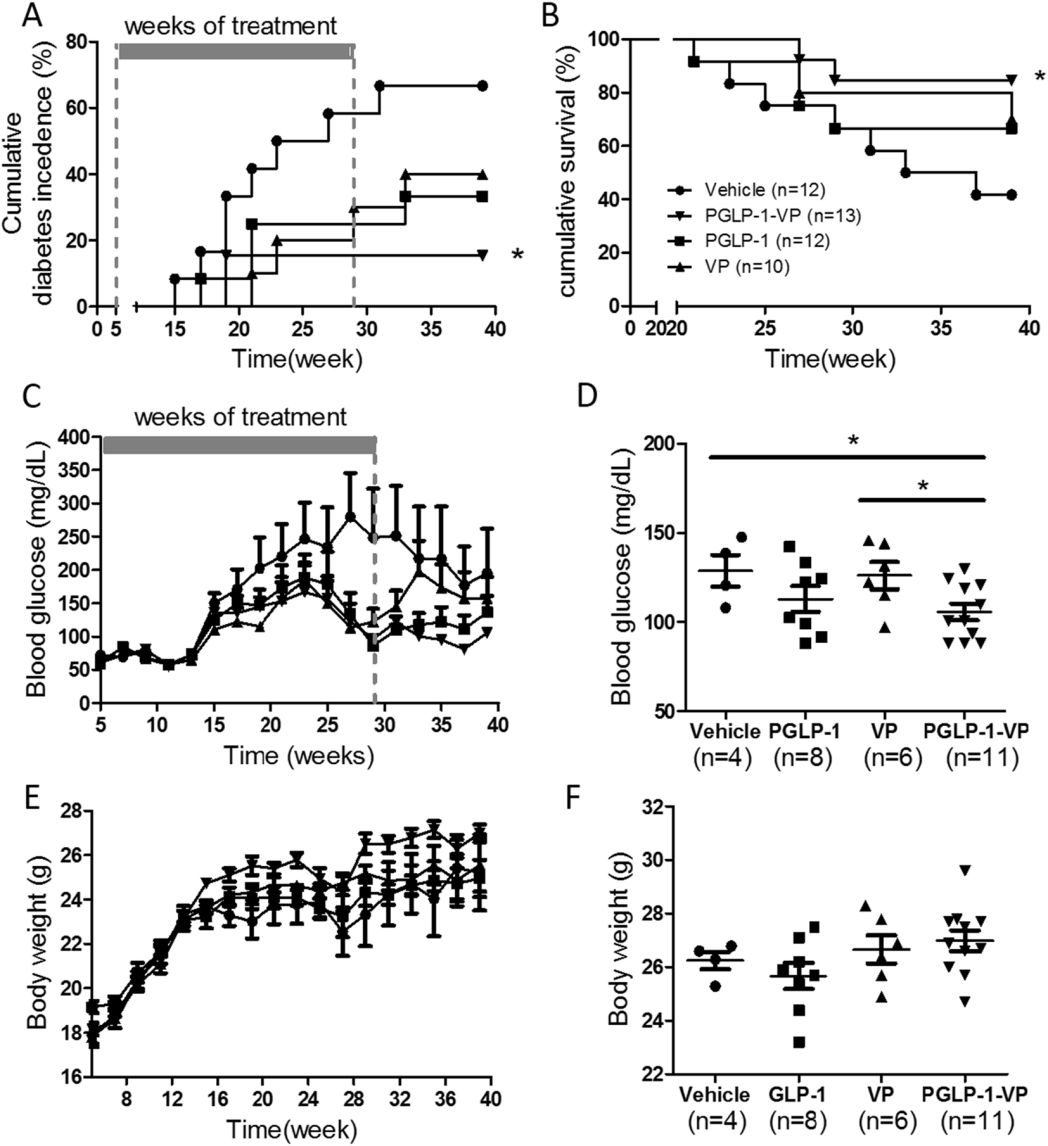
PGLP-1-VP treatment reduces morbidity and mortality in NOD mice. Cumulative incidence of diabetes (**A**) and cumulative survival rate (**B**) after 39 weeks of observation. Average blood glucose (**C**) and body weight (**E**) of each group. Week 39, individual blood glucose (**D**) and body weight (**F**) of every treatment group. *p < 0.05, PGLP-1-VP compared with vehicle.

Although PGLP-1-VP treatment was able to decrease fasting blood glucose in STZ-induced hyperglycemic mice, the effect of PGLP-1 in the NOD model is unknown. We therefore gave 5 week old female NOD mice with administration of either placebo, PGLP-1, VP or PGLP-1-VP for 24 weeks, and continued observation for up to 39 weeks. In the placebo group, NOD mice began to develop diabetes at 15 weeks; at the end of the observation period, the cumulative incidence of diabetes was 75% (Fig. 5A). The cumulative incidence rate of diabetes PGLP-1 or VP alone was 33% and 40%, respectively, with the onset of diabetes occurring later than Vehicle, at 17 and 21 weeks, respectively. Thus, PGLP-1 or VP alone appeared to delay or prevent the onset of diabetes. The treatment of PGLP-1-VP further reduced the cumulative incidence rate of diabetes to 15% (p = 0.0107, compared with vehicle) (Fig. 5B), with an onset time of diabetes at 19 weeks. We conducted the same experiment with 10-week-old NOD mice and obtained the expected results (Supplementary Fig. 1). Combined, these data suggested that PGLP-1-VP was able to dramatically inhibit the development of diabetes, while modestly delaying the onset for mice that do develop diabetes.

After 24 weeks of treatment, mice were observed for another 10 weeks. PGLP-1-VP recipients did not develop new cases of diabetes, while PGLP-1 or VP groups did (Fig. 5A). This suggests that PGLP-1-VP treatment may continue to inhibit the onset of diabetes. Additionally, glycemia values of group PGLP-1-VP remained at a low level starting from 29 weeks (Stop administration from this week). At week 39, glycemia values of group PGLP-1-VP was significantly lower than surviving control and VP group (p < 0.05) (Fig. 5C,D). Body weights were not affected by PGLP-1, VP or PGLP-1-VP treatments compared to controls (Fig. 5E,F).

### PGLP-1-VP therapy increases pancreas weight and prevents progression of insulitis

**Figure 6:**
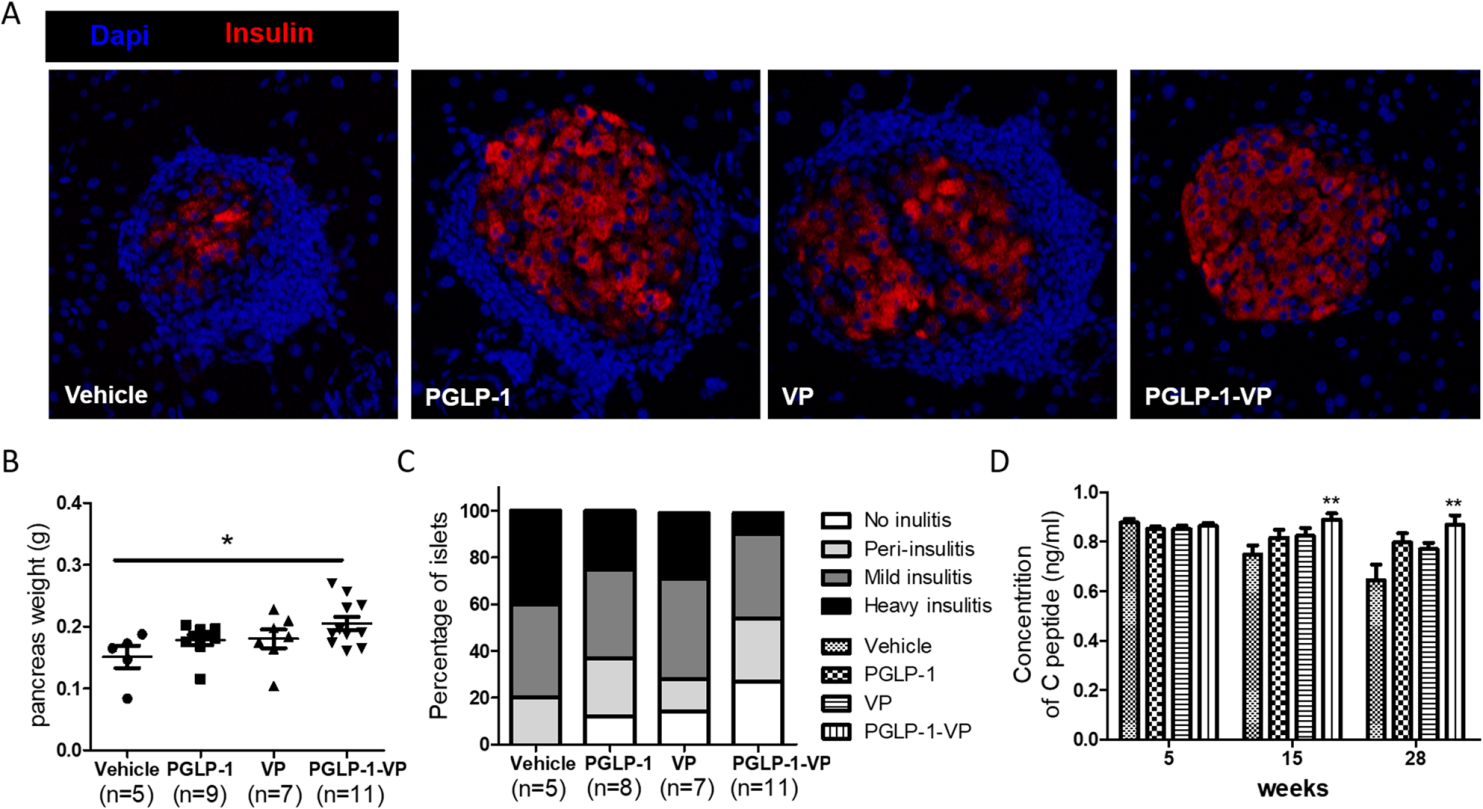
PGLP-1-VP treatment increases pancreas weight and prevents insulitis progression. (**A**) Pancreata were stained with anti-insulin (red) antibodies and visualized by fluorescence microscopy. (**B**) Pancreas weight of each group of mice after 39 weeks. (**C**) Pancreatic sections were taken from each group and blindly scored for islet inflammation. (**D**) The content of C peptide at week 15 and week 28. *p < 0.05, **p < 0.01, PGLP-1-VP compared with vehicle.

After 39 weeks, the mouse pancreas was taken, weighed, and subjected to haematoxylin and eosin staining and immunofluorescence staining. The results showed that the pancreas weight of the PGLP-1-VP treated group increased significantly (p < 0.05, compared with vehicle). Although PGLP-1 or VP treatment also increased pancreas weight, there was no significant difference (compared with vehicle) (Fig. 6A,B). Histological analysis showed that all mice from the vehicle group had insulitis, 40% of which were severe insulitis. PGLP-1, VP and PGLP-1-VP treatment were all able to reduce insulitis, and PGLP-1-VP treatment had the least insulitis (no insulitis, 27%; severe insulitis, 9% (Fig. 6C). Immunofluorescence staining of the pancreas also confirmed the PGLP-1-VP group had the least percentage of severe insulities (Fig. 6A).

At week 5, 15, and 28, we also tested the C-peptide content of the mice, and the results showed that the C-peptide content of the mice in the PGLP-1-VP-treated group was significantly increased (p < 0.01) (Fig. 6D). This indicates that PGLP-1-VP administration reduce the incidence of insulitis, increase pancreatic weight, and increase C-peptide content.

### PGLP-1-VP treatment corrects the imbalance of Th1 and Th2 cells

**Figure 7:**
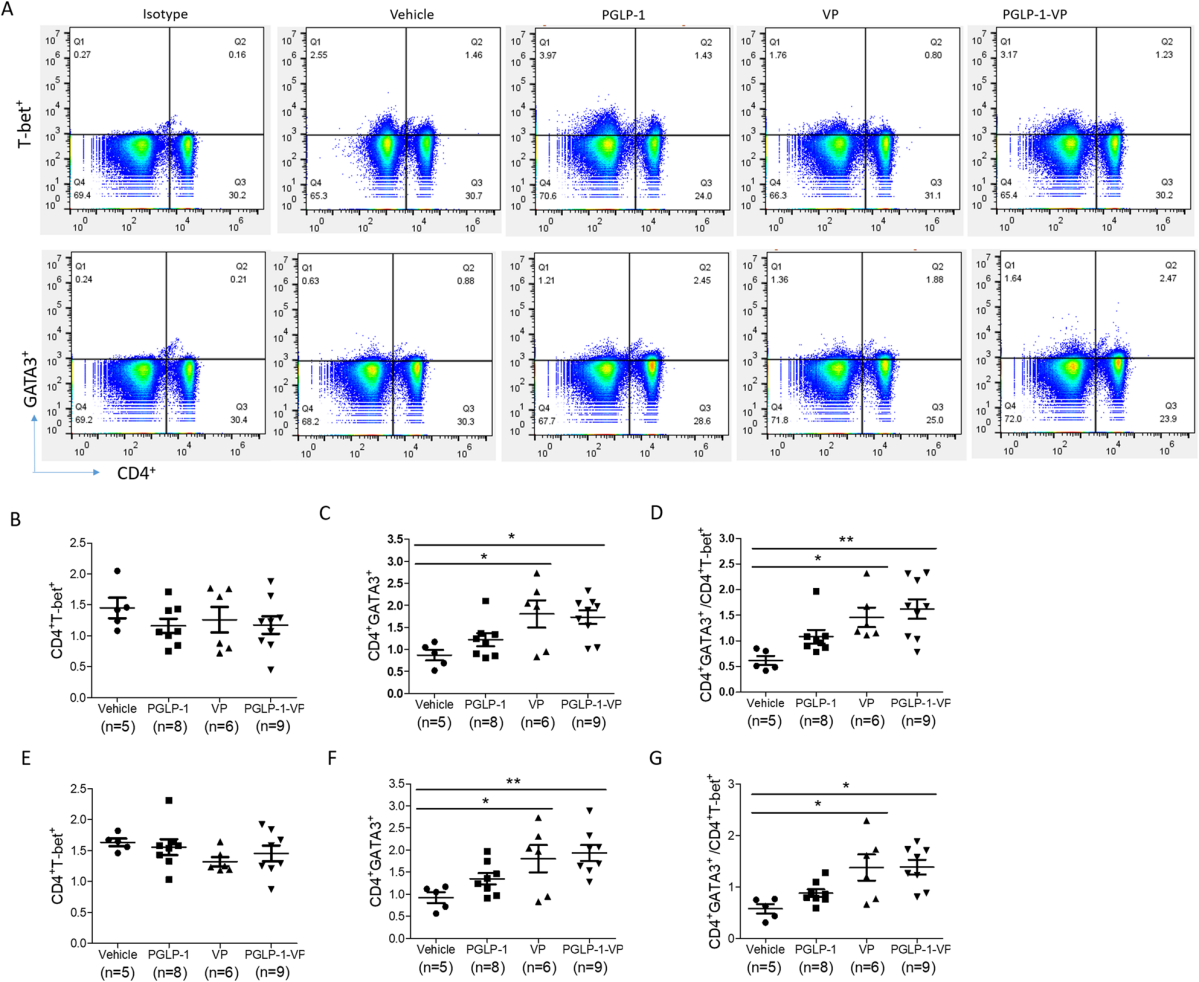
PGLP-1-VP treatment induces Th2 (GATA3^+^) cells in spleen and pancreatic lymph node (PLN). (**A**) Typical results of flow cytometry of each group. (**B**) Analysis of Th1 (T-bet^+^) cells by flow cytometry in spleen. (**C**) Analysis of Th2 cells by flow cytometry in spleen. (**D**) Analysis of Th2/Th1 values of each mouse in every group. (**E**) Analysis of Th1 (T-bet^+^) cells by flow cytometry in PLN. (**F**) Analysis of Th2 cells by flow cytometry in PLN. (**G**) Analysis of Th2/Th1 values of each mouse in PLN. *p < 0.05; **p < 0.01.

The interplay between Th1 and Th2 cells plays an important role in priming an immune response against pancreatic β cells^37–40^. In diabetic NOD mice, Th2/Th1 ratio is reduced due to an increase in Th1 cells and a decrease in Th2 cells^41^. Some literature reported that P277 can regulate the balance of Th1 and Th2 cells, and thus could play a role in the prevention of diabetes. Since VP and PGLP-1-VP are modified peptides of P277, we therefore analyzed Th1 and Th2 CD4^+^ T cells in the spleen and pancreatic lymph nodes of NOD mice. Our data showed revealed that VP and PGLP-1-VP treatment increased the percentage of Th2 cells and balanced the ratio of Th2/Th1 (Fig. 7A–G) in the spleen and pancreatic lymph node (PLN) of NOD mice. After PGLP-1-VP treatment, Th2 cells in spleen and PLN were 1.73% and 1.94%, respectively, significantly higher than the 0.87% and 0.92% in the control group (p < 0.05). The ratio of Th2/Th1 in spleen and PLN were 1.54% and 1.39%, respectively, significantly higher than 0.61% and 0.63% in the control group (p < 0.05). PGLP-1 treatment also increased Th2 cells, but had no significant difference. These data indicated that PGLP-1-VP was able to increase Th2 cells percentages and balanced the ratio of Th2/Th1 like P277 and VP.

### PGLP-1-VP treatment corrects the imbalance of Treg and Th17 cells

**Figure 8:**
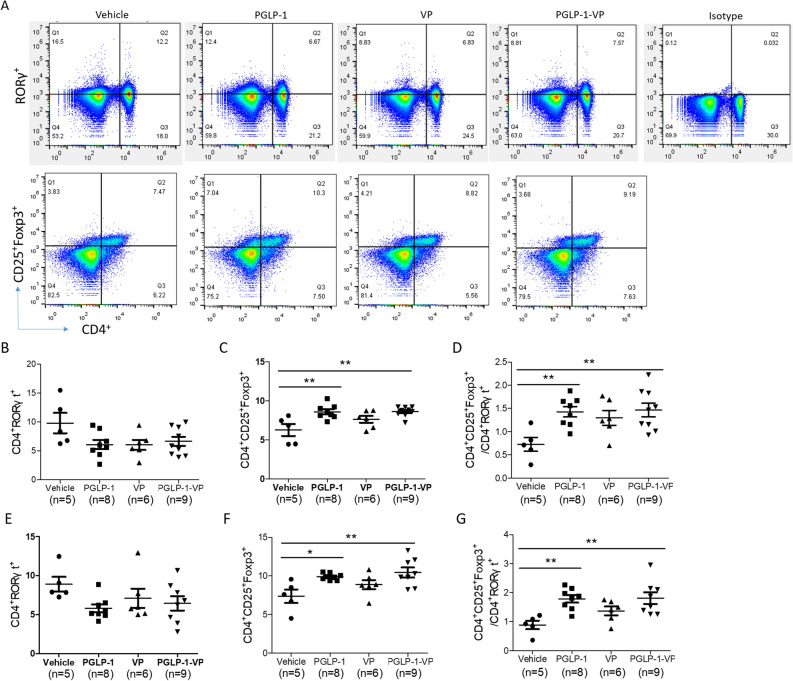
PGLP-1-VP treatment induces Treg (Foxp3^+^) cells in spleen and PLN. (**A**) Typical results of flow cytometry of each group. (**B**) Analysis of Th17 (RORγt ^+^) cells by flow cytometry in spleen. (**C**) Analysis of Treg cells by flow cytometry in spleen. (**D**) Analysis of Treg/Th17 values of each mouse in every group. (**E**) Analysis of Th17 (RORγt ^+^) cells by flow cytometry in PLN. (**F**) Analysis of Treg cells by flow cytometry in PLN. (**G**) Analysis of Treg/Th17 values of each mouse in PLN. *p < 0.05; **p < 0.01.

Because Treg cells have anti-inflammatory effects^37^ in type 1 diabetes, we examined the frequencies of Treg cells through flow cytometry. Foxp^3+^ Treg cells has higher frequencies in the spleens and PLN of NOD mice treated with PGLP-1 or PGLP-1-VP compared to vehicle group. The frequencies of Treg cells was 8.6% and 10.5% in the spleens and PLN of NOD mice treated with PGLP-1-VP, significantly higher than 6.3% and 7.4% in the control group (spleens, p < 0.01; PLN, p < 0.01) (Fig. 8A–G). Due to the important function of Th17 cells in autoimmune disease models^37^, RORγt^+^ Th17 cells were examined. The result revealed that PGLP-1 or PGLP-1-VP decreased Th17 cells, but had no significant difference. However, the ratio of Treg/Th17 increased significantly. The ratio of Treg/Th17 in PGLP-1-VP treatment group was 1.5% and 1.8% in the spleens and PLN, significantly higher than 0.7% and 0.8% in the control group (spleens, p < 0.05; PLN, p < 0.01) (Fig. 8A–G). Peptide VP treatment showed a trend for increasing Treg cells, decreasing Th17 cells, increasing the ratio of Treg/Th17 in the spleens and PLN, but there was no significant difference. All these data indicated that PGLP-1 or PGLP-1-VP was able to increase Treg cells and balanced the ratio of Treg/Th17, thereby preventing the occurrence of type 1 diabetes.

### PGLP-1-VP therapy increases TGF-β1 content

**Figure 9:**
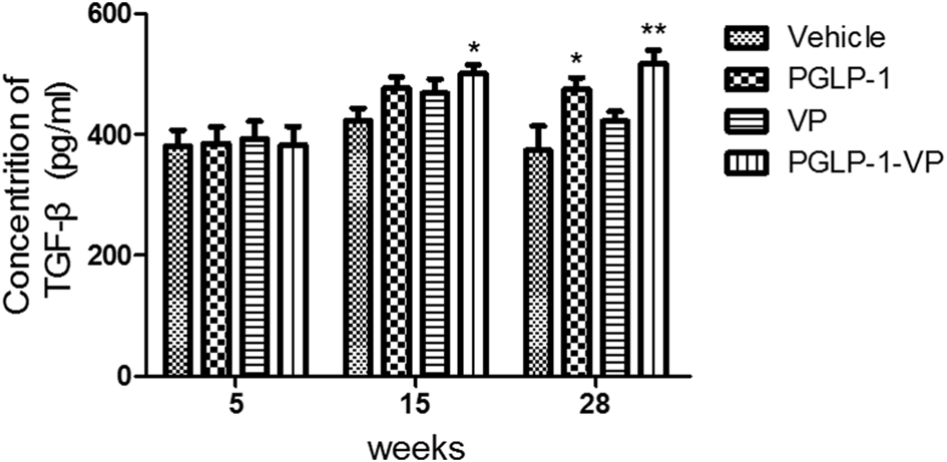
Plasma TGF-β1 concentrations determined by ELISA in NOD mice at week 5, week 15, and week 29. *p < 0.05; **p < 0.01.

Because TGF-β1 plays a key role in inhibiting islet autoimmune responses and improving autoimmune diabetes, we measured the level of TGF-β1 in the serum of NOD mice at week 5, week 15 and week 28. The results showed no difference in the levels of TGF-β1 in the serum of NOD mice at week 5, prior to treatment. At week 15 (10 weeks of administration), the serum TGF-β1 of the PGLP-1-VP group was significantly increased (p < 0.05, compared with vehicle), while no significant difference was found in PGLP-1 and VP groups (Fig. 9). At 29 week (24 weeks of treatment), the serum TGF-β1 of PGLP-1-VP group remained similar to Week 15, whereas the control group began to decline (PGLP-1-VP vs Vehicle, p < 0.01).

## Discussion

An effective T1DM therapy requires both the protection and regeneration of pancreatic β′ cells and the inhibition of autoimmune attacks. In our previous study, we showed that combining P277 with other proteins or peptides treatment resulted in a better therapeutic effect in NOD mice^31–34^. Peptide VP is a novel, modified P277 peptide that provides anti-diabetic effects without causing atherogenic side effects^30^ in NOD mice. Several human and animal studies have also demonstrated the potential role of GLP-1 RA in treating type 1 diabetes^17, 18^. We have previously demonstrated that PGLP-1 reduced blood glucose, increased β cell mass and inhibited weight loss in STZ-induced hyperglycemia mice^35^. Based on these existing results, we designed a dual-functional GLP-1-VP peptide, which is expected to act both as a GLP-1RA and as an immunomodulator. We demonstrated here that PGLP-1-VP, like native GLP-1, has the same affinity as GLP-1R, while being significantly more resistant to DPP-IV digestion in vitro*.* Additionally, IPGTT verified that PGLP-1-VP had comparable glucose-dependent insulinotropic action to GLP-1, but with a much longer half-life (more than 10 h effect to only 4 h). These data verified that PGLP-1-VP can effectively work as a GLP-1R agonist.

It was reported that GLP-1RA can reduce blood glucose, increase islet β cell mass and control body weight in animal model^14, 19, 42^, and some clinical trials have shown that GLP-1RA had beneficial effects in both new onset and longstanding T1DM patients^18, 43, 44^. Consistent with this, PGLP-1-VP treatment can significantly ameliorate hyperglycemia, reduce food intake and improve glucose and insulin tolerance as well as PGLP-1 in STZ-induced hyperglycemic mice model. However, PGLP-1-VP treatment did not inhibit body weight loss, in contrast to reports that PGLP-1 can inhibit body loss in STZ-induced hyperglycemic mice^35^. The discrepancy may be due to PGLP-1-VP completely resisting DPP-IV digestion (incubation with DPP-IV enzyme for 10 h in vitro showed almost no degradation), thereby has not insulinomimetic action like PGLP-1. All these indicated that PGLP-1-VP, as a GLP-1RA, can improve diabetes.

T1DM is a chronic autoimmune disease characterized by the destruction of insulin-producing β-cell in the pancreas. We next tested PGLP-1-VP treatment in the NOD mouse, an excellent autoimmune diabetes model containing many characteristics of human T1DM, including genetic risk factors, the presence of pancreas-specific autoantibodies, and autoreactive CD4^+^ T cells^45^. This model has been used extensively to test novel treatments for type 1 diabetes. Starting with 5-week-old NOD mice, 24 weeks of PGLP-1-VP treatment significantly reduced the cumulative incidence of diabetes, increased survival and delayed the onset of diabetes. Although treatment of PGLP-1 or VP alone also reduce the cumulative incidence of a disease, there was no significant difference compared with vehicle group. This indicated that PGLP-1-VP had the combined effects of PGLP-1 and VP. This may be associated with treatment with lighter insulitis and heavier pancreas in GLP-1-VP group. This also confirms that GLP-1RA can increase β cell mass^19^ and p277^34^ has immunoregulatory function. Collectively, our data suggested that PGLP-1-VP had the combined action of PGLP-1 and VP in the prevention and treatment of type 1 diabetes.

T1DM is thought to be caused by abnormal interactions between the genome, the immune system and the environment^46^. Pathogenesis of diabetes involves the dysregulation and dysfunction of T cells. In particular, islet antigen specific CD4^+^ T cells are key mediators in β cell damage and inflammatory injury^47^. Numerous studies have shown that alterations of Th1, Th2, Th17 cells, and Tregs play major roles in the etiology of T1DM^48^. Th1 cells exacerbates the development of T1DM while Th2 cells can ameliorate the autoimmune responses through producing cytokines such as IL-4 and IL-10^49^. In NOD mice, there is bias towards CD4 Th1 subtypes and a concomitant decrease in CD4 Th2 subtypes^50, 51^. In this study, we showed that PGLP-1-VP or VP treatment significantly increased Th2 cell percentage and elevated ratio of Th2/Th1 in spleen and PLN. However, there was no significant difference in Th1 cell percentage between the groups. These data indicated that PGLP-1-VP treatment was able to increase Th2 cells percentages and balanced the ratio of Th2/Th1 similarly to P277 and VP.

Islet autoreactive CD4^+^ T cells are normally deleted during thymic selection, and the few escapees are restrained in the peripheral by a variety of peripheral tolerance mechanisms. Immune tolerance mechanisms have been weakened in NOD mice, such that the slight disturbance between immune activation and tolerance can lead to the onset of the disease. Treg cells, the primary regulators of immune response and peripheral immune tolerance, play an important role in maintaining immune tolerance to islet antigens^52^. Some studies have shown that patients with T1DM have fewer and/or less effective Treg cells^53^. Herein, we first discovered that PGLP-1 or PGLP-1-VP, as a GLP-1R agonist, can increase Treg cells and enhance the ratio of Treg/Th17. This is consistent with the report that DPP-IV inhibitors can restore normoglycaemia^23, 24^ and improve islet graft survival^54^ through T-cell modulation in diabetic mice. These data indicated that PGLP-1-VP and PGLP-1 were able to suppress immune responses through increasing Treg cells. The production of immunosuppressive factor TGF-β1 is an important mechanism for Treg cells to suppress immune response ^52^, and TGF-β knockout (KO) mice show excessive activation during immune response. Here, we show that the levels of TGF-β1 in the serum of the PGLP-1-VP and PGLP-1 groups were significantly increased after 24 weeks of treatment compared to treatment onset. This is consistent with DPP-IV inhibitor treatment increasing TGF-β1 production in the plasma of NOD mice^23^. In contrast, serum TGF-β1 in the vehicle group after 24 weeks of treatment was comparatively lower than at treatment onset, suggesting that increased Treg cells may produce immunosuppressive effects by the production of TGF-β1. These results indicate that PGLP-1-VP can inhibiting islet autoimmune responses by increasing Treg cells like DPP-IV inhibitors.

T1D is one of the most common chronic diseases in children and also exists in adults^55–57^. The reason is that autoreactive T cells that evade central and peripheral immune tolerance destroy insulin-producing β cell^58^. Insulin therapy can save lives but requires daily use, increasing the risk of severe hypoglycemia, reducing but not avoiding other serious complications, including microvascular and macrovascular disease and death^59^. Usually, at the time of diagnosis, most T1DM patients have beta cells and still survive for 5 years^10–12^. Higher levels of endogenous insulin secretion are associated with lower rates of complications^60^, so there is a need for safe interventions to maintain or restore beta cell function, improve short- term and long-term outcomes. PGLP-1-VP can increase islet β-cell mass in STZ-induced hyperglycemia and NOD mice through activating GLP-1R, and reduce NOD mice morbidity, mortality, and pancreatic inflammation through anti-inflammation and immune regulation effects, which, as a dual-functional peptide, can improve glycemic control, reduce insulin usage, and avoid other serious complications in the prevention and treatment of type 1 diabetes.

As we all know, T1DM is a chronic T-lymphocyte-mediated autoimmune disease that can cause the destruction of pancreatic β-cells, which will develop in stages over time^61^. In the first stage, there is islet autoimmunity, but the blood sugar is normal; in the second stage, there is islet autoimmunity, and the blood sugar is abnormal, but there are no clinical symptoms; in the third stage, on the basis of the first two stages, there is clinical symptoms of hyperglycemia, polydipsia, polyuria and weight loss^62^. Drugs that can delay disease progression or improve microvascular or macrovascular complications will also be a useful supplement to the treatment options of T1DM patients. It is reported that GLP-1 RA has the potential to improve blood sugar control, reduce insulin dose and weight loss, without increasing the occurrence of hypoglycemia, and may be suitable as an additional therapy for insulin^18^. We combine the effects of GLP-1RA and P277 to regulate T cell immunity, reduce the incidence of NOD mice, and reduce the blood glucose level of mice. Therefore, we can treat TIDM in the early stage and hope to delay the onset of diabetes.

In summary, the findings described herein reveal that PGLP-1-VP may be a novel dual-functional peptide that functions as a GLP-1R agonist and as a VP peptide. The GLP-1R receptor binding, DPP-IV digestion stability, and glucose-dependent insulin secretion effects of PGLP-1-VP in vitro and in vivo verified that PGLP-1-VP is a long acting GLP-1R agonist. In STZ induced hyperglycemic mice model, PGLP-1-VP ameliorates STZ-induced hyperglycemia and improves insulin sensitivity, which indicates that PGLP-1-VP could work as a long-acting GLP-1R agonist in vivo. In the NOD mouse model, PGLP-1-VP as a dual-functional peptide is superior to PGLP-1 and VP alone in reducing morbidity, mortality, and pancreatic inflammation. Flow cytometry results showed that PGLP-1-VP can exert both the immunomodulatory function of VP peptide and the islet autoimmune inhibition function of GLP-1R agonist. Therefore, PGLP-1-VP may potentially act as a dual-functional peptide for anti-inflammation and immune regulation in the prevention and treatment of type 1 diabetes.

## Methods

### Peptide synthesis

GLP-1, VP, PGLP-1 and PGLP-1-VP were chemically synthesized by GL Biochem (Shanghai) Ltd. China.

### Human GLP-1 receptor activation

Human embryonic kidney (HEK-293) cells were obtained from ATCC and cultured in growing media (DMEM: 25 mM glucose, 10% FBS, 100 µg/mL penicillin and 100 µg/mL streptomycin). In our previous article, we utilized a co-transfection cell line CG-HEK293 cell which was able to express green fluorescence protein in response to the stimulation of GLP-1 or GLP-1 analogues^35^. GLP-1 receptor activation of PGLP-1-VP were tested and analyzed as previously described.

### Stability test for DPP-IV in vitro

GLP-1 or PGLP-1-VP (20 µM) were mixed with the same volume of 0.2 ng/µL rhDPP-IV enzyme (R&D Systems Inc. MN, USA.) and incubated different times at 37 ℃. Stability test for DPP-IV in vitro was performed as previously described^16^.

### Intraperitoneal glucose tolerance test (IPGTT) and insulin tolerance test (ITT)

Intraperitoneal glucose tolerance test was performed with 1.5 g/kg glucose in normal C57BL/6J mice (7–8 weeks old). Intraperitoneal glucose and insulin tolerance tests were performed with 1 g/kg glucose or 0.5 U/kg insulin, respectively, in STZ-induced hyperglycemic C57BL/6J mice.

### Mice and STZ in vivo treatment

All animal experiments were carried out in accordance with the National Institutes of Health guide for the care and use of laboratory animals (NIH Publications) as well as the ARRIVE guidelines and approved by the animal ethics committee of China Pharmaceutical University (Permit Number: 2162326). C57BL/6J mice (7–8 weeks old) were obtained from Model Animal Research Center of Nanjing University (Nanjing, China). Mice were single or group-housed on a 12-h/12-h light–dark cycle at 22 °C with free access to food and water.

Male C57BL/6J mice were injected intraperitoneally (i.p.) with 50 mg/kg body weight STZ (Sigma-Aldrich Inc. MO, USA) for 5 consecutive days^63, 64^. Blood glucose concentrations were measured at 7 and 14 days after the last injection of STZ, using a blood glucose meter (OMRON Healthcare (China) Co., Ltd. Dalian, China). Mice with two blood glucose measurements above 200 mg/dL were considered hyperglycemic.

### Glucose-lowering effect in STZ mice

The hyperglycemic mice were divided into 4 groups: vehicle, PGLP-1, VP and PGLP-1-VP, injected subcutaneously with placebo (vehicle, PBS) or 30 nmol/kg peptide for 4 weeks (twice a day). Fasting blood glucose and body weight (fasted 8 h) were assayed weekly. Food intake was measured every 3 or 4 days. Animals were euthanized by isoflurane overdose and dissected tissues were immediately frozen in liquid nitrogen, unless otherwise stated^16, 35^.

### Chronic treatments and follow-up in NOD mice

Female 4-week-old NOD mice were obtained from Beijing HFK Bioscience Co. Ltd. (Beijing, China). NOD mice were randomly divided into 4 groups: vehicle, PGLP-1, VP and PGLP-1-VP, and each group was subcutaneously injected twice weekly with placebo [Lipofundin^32^ (B. Braun Melsungen AG, Melsungen, Germany)] or 210 nmol/kg of peptides for 24 weeks. Body weight and blood glucose were measured once every 2 weeks until the end of the observation period. NOD mice were considered diabetic when blood glucose concentrations exceeded 200 mg/dL for 2 consecutive measurements. The onset time and death time of diabetic mice were calculated. Indexes of dead mice were shown as the last blood glucose or weight data. Blood samples were collected retro-orbital and centrifuged, and then serum was collected. Serum C-peptide levels and transforming growth factor-β1 (TGF-β1) were measured by enzyme-linked immunosorbent kits (Jiancheng Bio-Engineering Institute Co., Ltd. Nanjing, China).

### Pathology and histological examination

Pancreata were fixed in 4% paraformaldehyde overnight at 4 ℃ and embedded in paraffin blocks. Sections (7 μm thickness) were stained with hematoxylin and eosin for histological scoring of insulitis. Collect pancreatic sections at 100 µm intervals. Collect at least 25 islets for each pancreas sample to analyze the incidence and severity of insulitis^65^. Insulitis scoring was done by the pathologist who was blinded to the groups. Degrees of insulitis in the islets was scored as follows: No-insulitis (intact islets without inflammation); pre-insulitis (inflammation ≤ 25%); mild insulitis (25% < inflammation ≤ 50%); severe insulitis (50% < inflammation)^66^. Immunofluorescence staining was performed as previously described^35, 67^. Guinea pigs anti-mouse insulin (dilution 1:300; Abcam (Shanghai) Trading Co., Ltd. China) were used as the primary antibody. And the secondary antibodies used for immunofluorescence were donkey and goat anti-guinea pig IgG H&L (Alexa Fluor 488, Abcam (Shanghai) Trading Co., Ltd. China). The sections were examined using Olympus microscope and scanned in a systematic way using a computer with imaging software to control the data collection. The percentage of β-cell area per whole pancreas was calculated^35, 67^.

### Flow cytometry

Spleen cells and pancreatic lymphocytes were isolated from spleen and pancreatic lymph node (PLN) of 39-week-old NOD mice by mechanical disruption. Cells were respectively stained for Treg [(CD4^+^CD25^+^Foxp3^+^) (Foxp3 is a necessary factor for the development and function of Treg cells, and it is also the most sensitive marker for Treg cells)], Th17 (CD4^+^ Rorγt^+^), Th1 (CD4^+^ T-bet^+^) or Th2 (CD4^+^ GATA3^+^) phenotypes. The following antibodies were used: FITC anti-mouse CD4 (BioLegend Inc. CA, USA), PE anti-mouse CD25 (BioLegend Inc. CA, USA), APC anti-GATA3 (BioLegend Inc. CA, USA), Alexa Fluor 647 Mouse Anti-Mouse RORγt (BD Biosciences, CA, USA), Alexa Fluor 647 Mouse anti-T-Bet (BD Biosciences, CA, USA), Alexa Fluor 647 Rat anti-Mouse Foxp3 (BD Biosciences, CA, USA). Stained cells were detected by Flow cytometer (Accuri C6, BD Biosciences, CA, USA) and analyzed using BD Accuri C6 systerm Software.

### Statistical analysis

The data were expressed as means ± Standard Error of Mean (SEM) and analyzed using SPSS for windows version 13.0. Statistical significance was determined by Student’s t-test or one-way ANOVA followed by Student–Newman–Keuls post hoc test. The Kaplan–Meier curves were analyzed with Log-rank (Mantel-Cox) tests. Differences were considered statistically significant at p < 0.05.

## Supplementary Information

Supplementary Information.

## Abbreviations

GLP-1: Glucagon-like peptide-1
GLP-1R: Glucagon-like peptide-1 receptor
GLP-1RA: Glucagon-like peptide-1 receptor agonists
HbA1c: Glycosylated hemoglobin A1c
NOD mouse: Nonobese diabetic mouse
peptide VP: A ameliorated atherogenic side-effects P277 peptide
PGLP-1: A GLP-1 analogue
Th1 cell: T helper 1 cell
Th2 cell: T helper 2 cell
PLN: Pancreatic lymph node
STZ: Streptozotocin
DPP-IV: Dipeptidyl peptidase IV
HEK-293: Human embryonic kidney 293
IPGTT: Intraperitoneal glucose tolerance test
AUC: Area under the concentration time curve
ITT: Insulin tolerance test
TGF-β1: Transforming growth factor-β1
