# Supplementary material for: Continuous stimulation of dual-function peptide PGLP-1-VP inhibits the morbidity and mortality of NOD mice through anti-inflammation and immunoregulation
Source: Sci Rep. 2021 Feb 11;11:3593. doi: 10.1038/s41598-021-83201-4 (PMC7878925; doi:10.1038/s41598-021-83201-4)
Supplement: Supplementary file 1 — Supplementary Information. [file 41598_2021_83201_MOESM1_ESM.doc]

Continuous stimulation of dual-function peptide PGLP-1-VP inhibits the morbidity and mortality of NOD mice through anti-inflammation and immunoregulation

**Author**

Huashan Gao^1,2,†^, Qian Zhao^1,†^, Shanshan Tang^1^, Kaiying Li^1^, Fujian Qin^1^, Ziwei Song^1^, Yi Pan^1^, Liang Jin ^1,*^, Yanfeng Zhang^1,*^

1 State Key Laboratory of Natural Medicines, Jiangsu Key Laboratory of Drug Screening, School of life Science and Technology, China Pharmaceutical University, China;

2 College of Medicine, Pingdingshan University.

* Correspondence: Yanfeng Zhang ([zyf_3456@163.com](mailto:zyf_3456@163.com)); Liang Jin (ljstemcell@cpu.edu.cn),

† These authors contributed equally to this work


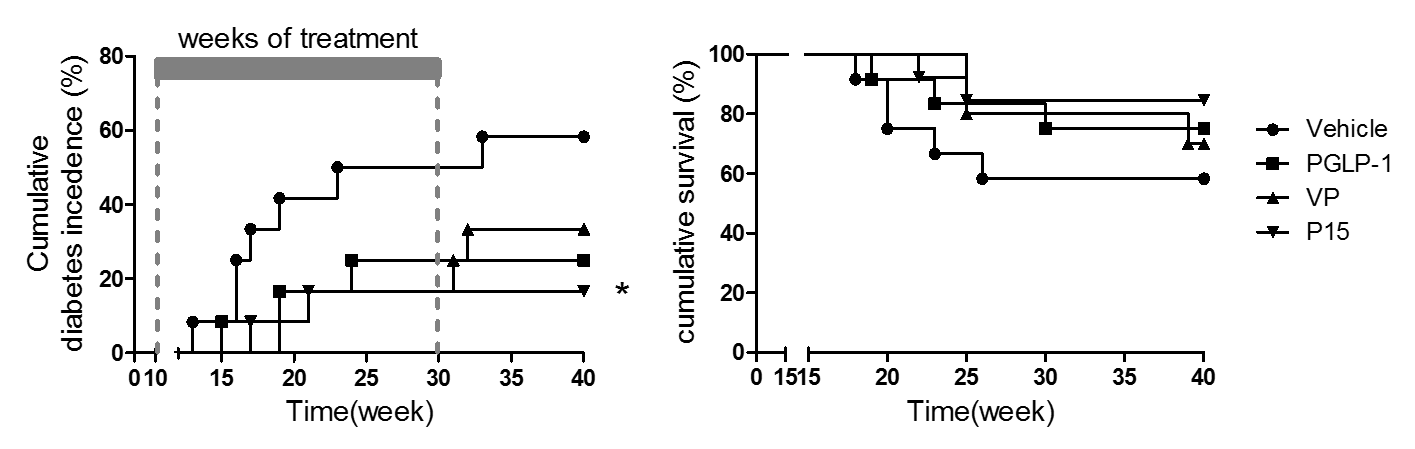


**Suppl. Fig. 1.** PGLP-1-VP treatment reduces morbidity and mortality in NOD mice. The NOD mouse mice (n=12) were administered at the age of 10 weeks, administered for 20 weeks, and the observation was stopped at 40 weeks. Cumulative incidence of diabetes (A) and cumulative survival rate (B) after 40 weeks of observation. *P < 0.05, PGLP-1-VP compared with vehicle.
